# Drinking While Stressed and Drinking to Cope Differentially Relate to Mental Health

**DOI:** 10.3390/bs14050402

**Published:** 2024-05-12

**Authors:** Carley Vornlocher, Michelle N. Shiota

**Affiliations:** Department of Psychology, Arizona State University, Tempe, AZ 85287-1104, USA; lani.shiota@asu.edu

**Keywords:** alcohol, coping, emotion regulation, stress, mental health

## Abstract

Excessive alcohol use, as well as drinking to manage distress, are known to undermine mental health. The current study examined the unique associations of simply consuming alcohol while stressed, versus using alcohol to cope with distress, with mental health during the early stages of COVID-19. Participants (*N* = 264) reported their alcohol use and use of alcohol/substances to cope with stress daily for 22 days and completed measures of mental health at baseline and every 7 days thereafter. Hierarchical regression models were tested to predict drinking while stressed, drinking to cope, and mental health. At baseline, drinking while stressed was not significantly associated with mental health when coping motives were accounted for. However, drinking to cope was significantly associated with greater stress, anxiety, depression, and loneliness. Drinking while stressed was significantly predicted by baseline stress and loneliness, while drinking to cope was significantly predicted by baseline anxiety. Drinking while stressed was not a significant predictor of change in mental health when coping motives were accounted for. Drinking to cope was a significant predictor of increases in depression but not of change in stress, anxiety, or loneliness. The motivation to manage distress appears to be driving the negative effects of alcohol use on mental health.

## 1. Introduction

Problematic alcohol use remains one of the leading preventable causes of death worldwide [1]. Thus, understanding what motivates alcohol use—and how health practitioners might intervene to mitigate alcohol use—remains an important, widely studied question. Various theoretical accounts have proposed that alcohol use is commonly facilitated by the motivation to regulate one’s emotions, especially in times of stress [2,3,4,5,6,7], though empirical evidence in support of these models is mixed. While strong evidence demonstrates that people consume more alcohol in response to experimentally induced negative affect/mood [8,9], this relationship appears to be less consistent in real-world contexts. In a meta-analysis of 69 studies employing daily diary or ecological momentary assessment protocols, no significant relationship between negative affect/mood and alcohol use was found, while alcohol use was positively associated with positive affect/mood [10].

Though the connection between stress and alcohol use remains unclear, widespread increases in alcohol use were observed during the early stages of the COVID-19 pandemic, and this was largely attributed to increases in stress [11,12,13]. Those struggling with their mental health appeared to be particularly susceptible to increases in drinking [12,14]. A meta-analysis of studies examining changes in alcohol use during COVID-19 across the globe found a relatively normal distribution of changes in alcohol use, such that across studies, approximately half of the participants reported no change in alcohol use, while approximately one-fourth reported increases, and one-fourth decreases, in alcohol use [15]. Stress and/or poor mental health were robust predictors of increased drinking behavior across studies.

Notably, these studies fail to disentangle simply drinking while stressed (DWS) from drinking with the distinct motive to cope with one’s stress (DTC). To differentiate these constructs, we define DTC as turning to alcohol with the explicit, intentional, conscious aim of reducing/coping with emotional distress. DWS, in contrast, is characterized by simply consuming alcohol concurrently with experiencing stress, not necessarily with an intentional coping/distress-reduction aim. Importantly, though theoretical models of coping and alcohol use suggest that DTC should be strongly predicted by stress, this does not seem to be the case. For those who report DTC, alcohol use does not appear to track fluctuations in affect/mood [10], suggesting that the motivation to cope via alcohol use may be consequential in its own right, regardless of how stressed one feels in the moment.

In theory, both DWS and DTC should be associated with diminished mental health, given the widely known negative effects of general alcohol use. Past work has shown that while DWS and DTC may sometimes boost mood at the moment, DTC particularly can prolong or exacerbate stress, independent from one’s drinking level [16,17,18], suggesting that disentangling these constructs is worthwhile. Moreover, coping motives have typically been examined as predictors of DWS or general alcohol use. Consequently, less is known about how DWS and DTC uniquely predict changes in mental health over time when both are accounted for in the same model. Disentangling the effects of DWS and DTC would provide important insight into how affective and behavioral processes contribute to both physical and mental health.

The current study seeks to examine how different facets of mental health relate to DWS and DTC. Analyses use archival data from a 22-day longitudinal study conducted during May–August of 2020, a year marked by record levels of stress, worry, sadness, and anger worldwide [19]. During the time frame of late spring through summer of 2020, the U.S. saw decreases in reported cases of COVID-19, government restrictions were being relaxed, communities were adapting to the restrictions that remained, and many were spending more time outside and beginning to safely reconnect with others. Nonetheless, U.S. adults were consistently reporting moderate levels of stress [20] and diminished mental health (i.e., depressive and anxious symptoms) [21] during this time period. This stress was attributable to a variety of sources beyond the risk of illness, including financial insecurity/access to basic needs, social isolation, and the sociopolitical climate of the U.S. at the time [22]. Though COVID-19 amplified these stressors during the spring–summer of 2020, they are not unique to the COVID-19 context and continue to affect people today [23]. Thus, we utilize this dataset not to demonstrate effects specific to the COVID-19 pandemic but effects likely to generalize to other contexts in which ongoing, chronic stress is experienced.

The use of 17 distinct coping strategies, as well as alcohol use, was assessed daily for 22 days. One of these strategies was drinking/substance use “to cope with COVID-19-related stress”; this was used as the measure of DTC in the present analyses. The number of drinks per day was used as the measure of DWS, with stress inferred from the broader situational context and normative experience in the U.S. at the time. A longer battery of measures assessing mental health was completed on the first day and every seventh day after. The current study focused on stress, anxiety, depression, and loneliness, given the widespread decreases in these facets of mental health at the time. While a growing body of research has now shown that people both drank more and experienced greater distress during this time frame, less is known about how these experiences were related to each other and how they were related to coping motives. To address these gaps in the literature, our core research questions assessed the relationships among participants’ daily reports of alcohol use and utilization of substances to cope during this period of ongoing stress, as well as weekly reports of subjective stress, anxiety, depression, and loneliness. Thus, we investigated three core questions:

Q1: Which facets of mental health are cross-sectionally related to DWS and DTC?

Q2: Do facets of mental health differentially predict DWS and DTC behavior?

Q3: Do DWS and DTC uniquely predict changes in mental health over time?

## 2. Materials and Methods

### 2.1. Transparency and Openness

The current manuscript presents secondary analyses that were not preregistered. The original study’s design was registered on OSF after the start of data collection, prior to data being analyzed. De-identified data used in the present analyses, as well as SPSS (version 29) and R (version 4.1.0) code and output, are available here: https://osf.io/37azc/ (accessed on 25 January 2024).

### 2.2. Participants

Participants (*N* = 264) were recruited from greater Phoenix, Arizona, and Los Angeles and San Francisco, California, via Craigslist and NextDoor advertisements. The sample was stratified with a target demographic breakdown of age evenly distributed across the adult lifespan; 60% White/European American, 15% Latinx, 15% Asian/Pacific Islander, and 10% Black/African American; 50% female; and an income distribution representative of the U.S. The final sample had a mean age of 41.65 (*SD* = 15.42), were 66.3% White, 10.2% Latino/a/x, 15.2% Asian, 10.0% Black, and 3.0% Native American, and 60.6% female and 39.0% male. For more details about the sample, please see Langley and colleagues (2023) or our OSF folder: https://osf.io/37azc/ (accessed on 25 January 2024).

### 2.3. Procedure

Those who were at least 18 years old and filled a needed demographic cell were invited to participate. Participants received an email invitation to the study, which included a link to the consent form and initial survey containing baseline measures of mental health, engagement in COVID-related health behaviors over the past week, personality, attachment style, and demographic information. Mental health measures were completed every seventh day from the baseline assessment. Participants also completed an adapted version of the Brief COPE [24,25], measures of general health behaviors (sugar intake, sleep, physical activity, nicotine use, and alcohol consumption), and two brief measures of relationship quality. This group of questions was completed daily for 22 days. This longitudinal, daily diary design allows for a nuanced assessment of changes in affect, behavior, and mental health over time. Participants were paid $60 via PayPal upon study completion. See Langley and colleagues (2023) for more details about study design and procedure [24].

### 2.4. Measures

#### 2.4.1. Drinking to Cope

The implementation of 17 strategies for coping with daily stress was assessed using a modified, 34-item version of the Brief COPE [24,25]. Participants rated the extent to which they utilized each strategy to deal with COVID-19-related stress in the past 24 h. Response options ranged from 1 (I didn’t do this at all) to 4 (I did this a lot). The current analyses consider the substance use subscale of this measure (α = 0.68–0.93). For each day, the two items (“I used alcohol or other drugs to help me get through” and “I used alcohol or other drugs to make myself feel better”) capturing the use of this strategy were averaged together to create a daily drinking to cope score. For certain analyses, an aggregate score reflecting drinking to cope across the entire 22-day study period was used; this score was created by averaging responses to these items across the 22 daily reports.

#### 2.4.2. Drinking While Stressed

Participants reported daily on the number of servings of alcohol (1 oz liquor, 5 oz wine, or 12 oz beer) they consumed in the past 24 h. Response options included the following: 0 (0 servings), 1 (1 serving), 2 (2 servings), 3 (3 servings), 4 (4 servings), or 5 (5 or more servings). Though participants were not explicitly asked to report if they were stressed while drinking, we make the reasonable assumption that most participants were at least moderately stressed throughout the duration of the study, given the findings outlined in the Introduction [19,20,21,22,23].

#### 2.4.3. Emotional Distress

The DASS-21 [26] is a 21-item measure of emotional distress across three categories: depression (α = 0.90–0.91; e.g., “I couldn’t seem to experience any positive feeling at all”), anxiety (α = 0.84; e.g., “I felt I was close to panic”), and stress (α = 0.86–0.88; e.g., “I found it difficult to relax”). Items were phrased to assess how participants felt over the past week, with response options ranging from 1 (not at all) to 4 (most of the time). Responses to the seven items reflecting each subscale were summed and then multiplied by 2 to create a final subscale score. Higher scores on any subscale are indicative of higher emotional distress.

#### 2.4.4. Loneliness

The 20-item UCLA Loneliness Scale [27] was used to measure participants’ self-reported feelings of loneliness and social isolation (α = 0.51–0.62). Items were phrased to assess how often participants felt the way described by each item over the past week, with response options ranging from 1 (never) to 4 (often). Items were averaged together, with higher scores indicating greater loneliness.

## 3. Results

A full breakdown of descriptive statistics for key variables can be found in Table 1. The Pearson correlation of drinking while stressed (*M* = 0.55, *SD* = 0.86) with drinking to cope (*M* = 1.45, *SD* = 0.69) across the 22-day study period was positive, strong, and significant, *r* = 0.74, *p* < 0.001.

Hierarchical regression models were used to examine the relations among drinking while stressed, drinking to cope, and mental health outcomes. First, a series of hierarchical regression models were tested, with baseline (Day 1) mental health outcomes regressed on baseline drinking while stressed and drinking to cope. Gender, age, and income were entered in Step 1 as control variables. Drinking while stressed was entered in Step 2. Drinking to cope was entered in Step 3. The results of these models are presented in Table 2. In the models that included only control variables and baseline drinking while stressed, drinking while stressed was significantly positively related to baseline stress (β = 0.17, *p* < 0.01), anxiety (β = 0.21, *p* < 0.001), and depression (β = 0.17, *p* < 0.01), but was not related to loneliness (β = 0.05, *p* = 0.42). However, in the final model, including both baseline drinking while stressed and drinking to cope, drinking while stressed was not significantly related to baseline stress (β = −0.05, *p* = 0.49), anxiety (β = −0.01, *p* = 0.89), depression (β = −0.04, *p* = 0.57), or loneliness (β = −0.13, *p* = 0.08). Baseline drinking to cope was significantly positively related to baseline stress (β = 0.36, *p* < 0.001), anxiety (β = 0.36, *p* < 0.001), depression (β = 0.35, *p* < 0.001), and loneliness (β = 0.29, *p* < 0.001). A full summary of these models can be found in Table 2.

Next, a series of hierarchical regression models were tested to examine if baseline mental health would predict drinking while stressed and drinking to cope over the 22-day study period. Gender, age, and income were entered in Step 1 as control variables. Each mental health measure was then entered in a separate step of the model. Drinking while stressed was significantly positively predicted by baseline stress (β = 0.19, *p* < 0.05) and significantly negatively predicted by baseline loneliness (β = −0.17, *p* < 0.05) but not related to baseline anxiety (β = 0.01, *p* = 0.37) or depression (β = 0.01, *p* = 0.20). Drinking to cope was significantly positively predicted by baseline anxiety (β = 0.26, *p* < 0.01) but not related to baseline stress (β = 0.13, *p* = 0.16), depression (β = 0.14, *p* = 0.11), or loneliness (β = −0.08, *p* = 0.31). Notably, males (*M* = 0.70, *SD* = 0.94) reported significantly greater drinking while stressed than females (*M* = 0.44, *SD* = 0.81), β = −0.18, *p* < 0.01. Males (*M* = 1.56, *SD* = 0.67) also reported drinking to cope to a greater extent than did females (*M* = 1.38, *SD* = 0.68), β = −0.15, *p* < 0.05. A full summary of these models can be found in Table 3.

Next, a series of hierarchical regression models were tested to examine if drinking while stressed and drinking to cope over the 22-day study period would predict changes in mental health from Week 1 to Week 4. Gender, age, and income were entered in Step 1 as control variables. Baseline mental health was then entered in Step 2. Drinking while stressed was entered in Step 3. Drinking to cope was entered in Step 4. In the models that included only control variables, baseline mental health, and drinking while stressed, drinking while stressed was a significant positive predictor of increases in anxiety (β = 0.13, *p* < 0.01) and depression (β = 0.15, *p* < 0.01). However, in the final model, including both drinking while stressed and drinking to cope, drinking while stressed was not a significant predictor of change in stress (β = −0.01, *p* = 0.91), anxiety (β = 0.04, *p* = 0.61), depression (β = 0.03, *p* = 0.65), or loneliness (β = 0.00, *p* = 0.97). Drinking to cope was a significant predictor of increases in depression (β = 0.17, *p* < 0.05) but not of change in stress (β = 0.12, *p* = 0.09), anxiety (β = 0.14, *p* = 0.07), or loneliness (β = 0.04, *p* = 0.57). A full summary of these models can be found in Table 4.

Given the strong, positive correlation between drinking while stressed and drinking to cope, we conducted an exploratory probe of the interaction between drinking while stressed and drinking to cope in predicting mental health outcomes. Notably, drinking to cope did not moderate the effect of drinking while stressed on any mental health outcome, and drinking while stressed did not moderate the effect of drinking to cope on mental health either (*p* values = 0.41–0.67).

## 4. Discussion

The current study broadly assessed how different facets of mental health were related to drinking while stressed and drinking to cope with one’s stress. Despite evidence demonstrating that DWS and DTC are distinct [10], much prior literature has conflated these behaviors. The present study teased apart the effects of DWS and DTC by exploring these phenomena in the context of COVID-19, a period during which most people were experiencing significant distress and attempting to cope. Across models, drinking while stressed was generally associated with diminished mental health until drinking to cope was accounted for; when coping behavior was included in the models, the effects of drinking while stressed were no longer significant. In contrast, drinking to cope was a rather consistent predictor of diminished mental health, over and above the effects of drinking while stressed. Notably, these effects emerged even though most of our sample did not drink heavily—if they drank at all.

Drinking while stressed—absent of motives to cope—was not deleterious to mental health. Drinking while stressed was not associated with mental health at baseline, nor did drinking while stressed predict changes in mental health over time when coping behavior was accounted for in the same models. Moreover, higher baseline stress was associated with greater DWS during the 22-day study period, while those who were lonelier were less likely to DWS. These findings suggest that while those who report experiencing greater stress may be more likely to drink, drinking while stressed does not necessarily exacerbate one’s emotional distress over the long term. Although it is reasonable to assume that drinking while stressed would still pose physical health costs, such costs may be the result of direct physiological effects of drinking while stressed rather than effects via diminished mental health.

Our findings are in line with past work demonstrating that drinking to cope is not reliably predicted by emotional distress [10]. Though anxiety did predict the tendency to DTC, stress, loneliness, and depression did not. These findings contradict past theoretical accounts explaining drinking behavior [2,3,4,5,6,7] and suggest that DTC is not triggered primarily by one’s situational context but rather by a dispositional tendency to engage in such behaviors. What might explain this dispositional tendency? Perhaps those who drink to cope show a more general tendency to utilize emotion-focused coping strategies, seeking emotional comfort rather than taking steps to improve their situation and/or appraising/thinking about the situation in ways that make it less distressing. Such strategies have been shown to be less effective at improving mental health than strategies that intervene earlier on in the emotion process [28,29,30,31], which may explain why DTC, rather than DWS, is the more robust predictor of mental health. Critically, there was no observed interaction between DWS and DTC, such that the relationship between DTC and mental health outcomes was not qualified by how much participants drank while stressed. This null effect further underscores that the links between consuming alcohol to cope with stress/distress and diminished mental health are not explained well by alcohol use per se but rather by some aspect of the psychological process driving alcohol use when under stress.

One important possibility to consider is that DWS and DTC may merely be picking up on individual differences in awareness of one’s thoughts, feelings, and behaviors. It is plausible that the observed differences between DWS and DTC are not actually driven by different motives/behaviors per se but rather by awareness of such motives/behaviors. If this were the case, however, we would expect to have seen the opposite pattern of effects, such that those who are aware of their motives/behaviors (and report DTC) would actually report greater mental health than those unaware of their own motives/behaviors (and report DWS). Future work might explore this further by using alternative methods of measuring drinking to cope.

Our findings suggest that health practitioners seeking to improve the mental health of those who do use alcohol would see the most benefit from mitigating the motive to use alcohol *as a coping tool*. If coping is motivating alcohol use, broadening people’s emotion regulation toolkits by providing training in alternative coping strategies may prove useful in breaking the link between diminished mental health and alcohol use. However, future work is needed to investigate which specific emotion regulation strategies would be useful—and well-accepted—alternatives for those who tend to rely on alcohol to cope.

The current study utilized data collected from a diverse sample using a longitudinal design, allowing for the examination of both cross-sectional associations and predictors of change in mental health over time. Though our findings provide valuable insights into the unique effects of DWS and DTC on mental health, it is unclear if such effects are unique to those experiencing chronic stress/mental illness or if these differential effects may translate to other contexts. Moreover, our study did not account for other possible motives for drinking behavior— especially those relating to affective processes. For example, future work should explore how enhancement motives—drinking to boost positive mood—relate to coping motives and how these motives contribute to mental health outcomes, especially in the context of stress. Past work has shown that emotion regulation strategies that aim to foster pleasant experiences and/or positive emotions in the face of stress can be adaptive for mental health [32,33,34]. Perhaps using alcohol to boost positive affect in spite of stress may show similar benefits.

Moreover, a core takeaway from the current study is that DWS and DTC are distinct behaviors. Though these two constructs were strongly correlated, it is unclear what exactly causes people to DWS if they are not using alcohol to cope with their stress. Given that stress taxes our capacity for self-regulation [35], DWS may be explained by an inability to resist temptation in the presence of alcohol if others are drinking or even a reversion to habitual behaviors [36], which may or may not be ideal. Past work has considered impaired control as an important predictor of problematic drinking behaviors [37]; future work should explore the role of impaired control in the context of ongoing stress and how impaired control may explain certain drinking behaviors.

### Significance

The current study refines our understanding of how drinking behavior relates to mental health by integrating perspectives from affective and health sciences. While past work has tended to conflate simply drinking while stressed and drinking with the distinct motive to change one’s current emotional state, we demonstrate that these behaviors have distinct implications for mental health outcomes. Further, our findings highlight drinking to cope as an important point of intervention for improving mental health outcomes. Health practitioners may be able to enhance the mental health of those who experience stress by mitigating the desire to use alcohol as a coping tool and/or providing alternative strategies for coping with stress. 

## Figures and Tables

**Table 1:** Descriptive statistics of key variables.

	α	*M (SD)*
Week 1 Stress	0.86	30.73 (9.88)
Week 4 Stress	0.88	29.54 (10.21)
Week 1 Anxiety	0.84	22.79 (8.63)
Week 4 Anxiety	0.84	21.77 (8.36)
Week 1 Depression	0.91	28.21 (10.89)
Week 4 Depression	0.90	27.45 (10.76)
Week 1 Loneliness	0.51	2.10 (0.68)
Week 4 Loneliness	0.62	2.09 (0.68)
Day 1 Drinking While Stressed	N/A	0.39 (0.95)
Day 1 Drinking to Cope	0.93	1.61 (0.92)
Aggregated Drinking While Stressed (Days 1–22)	0.96	0.55 (0.86)
Aggregated Drinking to Cope (Days 1–22)	0.99	1.45 (0.69)

**Table 2:** Hierarchical regression models with baseline mental health regressed on baseline drinking while stressed and drinking to cope.

	Stress	Anxiety	Depression	Loneliness
Model 1				
R^2^ Change	0.11 ***	0.06 **	0.09 ***	0.12 ***
Gender	0.15 *	0.05	0.10	0.05
Age	−0.30 ***	−0.24 ***	−0.27 ***	−0.30 ***
Income	−0.04	−0.05	−0.10	−0.18 **
Model 2				
R^2^ Change	0.03 **	0.04 ***	0.03 **	0.00
Gender	0.17 **	0.08	0.12 *	0.06
Age	−0.30 ***	−0.24 ***	−0.27 ***	−0.29 ***
Income	−0.04	−0.05	−0.10	−0.18 **
Drinking While Stressed	0.17 **	0.21 ***	0.17 **	0.05
Model 3				
R^2^ Change	0.08 ***	0.08 ***	0.08 ***	0.05 ***
Gender	0.17 **	0.08	0.12 *	0.06
Age	−0.25 ***	−0.20 ***	−0.23 ***	−0.26 ***
Income	−0.03	−0.03	−0.08	−0.16 **
Drinking While Stressed	−0.05	−0.01	−0.04	−0.13
Drinking to Cope	0.36 ***	0.36 ***	0.35 ***	0.29 ***

Standardized regression coefficients are presented here. * indicates *p* < 0.05, ** indicates *p* < 0.01, and *** indicates *p* < 0.001. Gender was coded such that 1 indicates male and 2 indicates female.

**Table 3:** Hierarchical regression models with drinking while stressed and drinking to cope over the 22-day study period regressed on baseline mental health.

	Drinking While Stressed	Drinking to Cope
Model 1		
R^2^ Change	0.03	0.04 *
Gender	−0.14 *	−0.12
Age	−0.06	−0.15 *
Income	0.06	−0.04
Model 2		
R^2^ Change	0.06 ***	0.12 ***
Gender	−0.18 **	−0.16 **
Age	0.02	−0.04
Income	0.07	−0.03
Stress	0.25 ***	0.36 ***
Model 3		
R^2^ Change	0.00	0.04 ***
Gender	−0.18 **	−0.15 *
Age	0.02	−0.03
Income	0.07	−0.02
Stress	0.20 *	0.18 *
Anxiety	0.07	0.27 ***
Model 4		
R^2^ Change	0.00	0.01
Gender	−0.18 **	−0.15 *
Age	0.02	−0.03
Income	0.08	−0.01
Stress	0.18	0.12
Anxiety	0.06	0.25 **
Depression	0.03	0.10
Model 5		
R^2^ Change	0.02 *	0.00
Gender	−0.18 **	−0.15 *
Age	0.00	−0.04
Income	0.06	−0.02
Stress	0.19 *	0.13
Anxiety	0.08	0.26 **
Depression	0.12	0.14
Loneliness	−0.17 *	−0.08

Standardized regression coefficients are presented here. * indicates *p* < 0.05, ** indicates *p* < 0.01, and *** indicates *p* < 0.001. Gender was coded such that 1 indicates male and 2 indicates female.

**Table 4:** Hierarchical regression models predicting change in mental health from drinking while stressed and drinking to cope across 22 days.

	Stress	Anxiety	Depression	Loneliness
Model 1				
R^2^ Change	0.07 ***	0.04 *	0.04 *	0.07 ***
Gender	0.16 *	−0.01	0.10	0.06
Age	−0.21 ***	−0.19 **	−0.09	−0.16 **
Income	−0.04	−0.05	−0.15 *	−0.20 ***
Model 2				
R^2^ Change	0.41 ***	0.39 ***	0.42 ***	0.48 ***
Gender	0.06	−0.04	0.03	0.02
Age	−0.01	−0.04	0.10 *	0.05
Income	−0.01	−0.02	−0.08	−0.07
Baseline Mental health	0.68 ***	0.64 ***	0.68 ***	0.74 ***
Model 3				
R^2^ Change	0.01	0.02 **	0.02 **	0.00
Gender	0.07	−0.02	0.05	0.03
Age	−0.01	−0.04	0.10 *	0.06
Income	−0.02	−0.02	−0.09 *	−0.08
Baseline Mental health	0.66 ***	0.62 ***	0.65 ***	0.74 ***
Drinking While Stressed	0.07	0.13 **	0.15 **	0.03
Model 4				
R^2^ Change	0.01	0.01	0.01 *	0.00
Gender	0.07	−0.02	0.06	0.03
Age	−0.01	−0.03	0.11 *	0.06
Income	−0.01	−0.01	−0.08	−0.07
Baseline Mental health	0.64 ***	0.58 ***	0.62 ***	0.73 ***
Drinking While Stressed	−0.01	0.04	0.03	0.00
Drinking to Cope	0.12	0.14	0.17 *	0.04

Standardized regression coefficients are presented here. * indicates *p* < 0.05, ** indicates *p* < 0.01, and *** indicates *p* < 0.001. Gender was coded such that 1 indicates male and 2 indicates female.

## Data Availability

De-identified data and analytical code used in the present analyses are available on OSF: https://osf.io/37azc/ (accessed on 25 January 2024).
